# The Effect of Combined Therapy ICS/LABA and ICS/LABA plus Montelukast in Patients with Uncontrolled Severe Persistent Asthma Based on the Serum IL-13 and FEV1

**DOI:** 10.3889/oamjms.2015.053

**Published:** 2015-05-07

**Authors:** Elena Jovanovska Janeva, Zlatica Goseva, Angjelko Gjorchev, Angela Debreslioska, Mirko Spiroski, Beti Zafirova, Magdalena Genadieva Dimitrova

**Affiliations:** 1 *University Clinic of Pulmonology and Allergology, Faculty of Medicine, Ss Cyril and Methodius University of Skopje, Skopje, Republic of Macedonia*; 2 *Institute of Immunobiology and Human Genetics, Faculty of Medicine, Ss Cyril and Methodius University of Skopje, Skopje, Republic of Macedonia*; 3 *Institute of Epidemiology and Biostatistics, Faculty of Medicine, Ss Cyril and Methodius University of Skopje, Skopje, Republic of Macedonia*; 4 *University Clinic of Gastroenterohepatology, Faculty of Medicine, Ss Cyril and Methodius University of Skopje, Skopje, Republic of Macedonia*

**Keywords:** asthma, IL-13, FEV1, ICS/LABA, Montelukast

## Abstract

**BACKGROUND:**

IL-13 is one of many cytokines responsible for the chronic inflammation of asthma.

**AIM:**

The aim of this study was to determine the effect of combined therapy ICS/LABA and ICS/LABA plus Montelukast in patients with uncontrolled severe persistent asthma by analyzing of serum IL-13 and FEV1 before the treatment and after 6 months of therapy.

**MATERIAL AND METHODS:**

In study we included two groups. First group with 27 patients were treated with ICS/LABA. Second group with 29 patients were treated with ICS/LABA plus Montelukast. In each of them were measured serum IL-13 levels by the ELISA method and FEV1 before and after 6 months of treatment. Results were statistically analyzed according to the Wilcoxon Pairs Test and T-test.

**RESULTS:**

The obtained results in both groups showed that the serum IL-13 before the start of therapy were much higher and after 6 months of treatment significantly reduces their value, which in the second group were more expressed. The difference in the average value of FEV1 in both groups before and after therapy was statistically significant.

**CONCLUSION:**

Treatment with ICS/LABA plus Montelukast proved superior compared to therapy of ICS/LABA in patients with uncontrolled severe persistent asthma and allows achievement of well controlled of asthma with subjective clinical improvement.

## Introduction

If the 20-th century was underlined with an “explosion” of allergic diseases and asthma, the 21-th century is already called epidemic century for allergic diseases and asthma. Asthma is a worldwide problem and also is one of the most common chronic diseases worldwide —300 millions or 7.2% patients suffered from asthma. Prevalence was increasing in many countries, especially in children — 6% and in adult 10%. There are 250,000 deaths annually. It is estimated that the number of people with asthma will grow by more than 100 million by 2025 [1-4]. In the Republic of Macedonia 100,000 or 5% of the population suffer from asthma [5, 6].

Asthma is a chronic inflammatory disorder of the airways in which many cells and cellular elements play a role (mast cells, eosinophils, T lymphocytes, macrophages, neutrophils, and epithelial cells). These mediators act on cells in the airway, leading to contraction of smooth muscle, oedema due to plasma leakage and mucus plugging. The chronic inflammation is associated with airway hypersponsiveness that leads to recurrent episodes of wheezing, breathlessness, chest tightness and coughing, particularly at night or in the early morning. These episodes are usually associated with widespread, but variable, airflow obstruction within the lung that is often reversible either spontaneously or with treatment [1, 7, 8].

In this chronic inflammation are involve more than 100 mediators and the Interleukins take central place in this inflammation [9, 10]. The interleukins are cytokines that stimulate the proliferation and differentiation of immune cells. IL-1 activates T cells, IL-2 stimulates the proliferation of antigen-activated T and B cells; IL-4, IL-5 and IL-6 stimulated proliferation and differentiation of B cells; interferon-gamma (IFNγ) activates macrophages while IL-3, IL-7, and (GM-CSF) stimulate hematopoiesis [11, 12].

T cells play a key role in coordinating the immune response in asthma. The key to the functioning of T cells is a molecule that binds to the antigen: T cells receptor. Generally the T cells which have the CD4 + act as helper cells (Th2-Ly), and CD8+ act as cytotoxic cells (Tc-Ly). CD4 + helper cells, differentiate into subpopulations of T cells in Th1, Th2, Th9, Th17, Th22 and T follicular effectors cell. Th2 cells produce, IL-4, IL-5, IL-9, IL-13, GM-CSF and IL-25, IL-31, IL-33 that are responsible for chronic eosinophilic inflammation, inflammation in allergic diseases, including and asthma [13, 16].

Interleukin 13 (IL-13) is one of many cytokines responsible for the chronic inflammation of asthma. IL-13 is synthesized by activated Th2 CD4 and CD8 cells in response to antigen specific stimulus. It has a similar function as IL-4. This is due to the ability to connect to a common receptor IL4R [16, 17].

Patients with asthma who have increase values of serum IL-13, lead to airway hyperresponsiveness, mucus hypersecretion, activation of fibroblasts and hyperplasia and hypertrophy of smooth muscle of the airways and if they do not use preventive anti-asthma treatment, may cause irreversible airway remodelling [18].

The National Asthma Education and Prevention Program have classified asthma as: intermittent, mild persistent, moderate persistent and severe persistent. These classifications are based on severity, which is determined by symptoms and lung function tests. You should be assigned to the most severe category in which any feature occurs [19].

Asthma is considered severe persistent if without treatment any of the following are true [19]. Symptoms: occur throughout each day, severely limit daily physical activities, night time symptoms occur often, sometimes every night, lung function tests are abnormal (FEV1<60% or less of expected value), and Peak Expiratory flow (PEF) varies more than 30% from morning to afternoon.

FEV1 - Forced expiratory volume in the first second is the maximal volume of air exhaled in the first second of a forced expiration from a position of full inspiration expressed. FEV1 should be measured from a series of at least three forced expiratory curves that have an acceptable start of test and are free from artefact, such as a cough. The largest FEV1 should be recorded after examining the data from all of the usable curves, even if they do not come from the same curve. The calculated values than are converted to a percentage of normal, based on date of birth, height, weight, gender and race. FEV1 is a marker for the degree of airway obstruction in asthma. Reversibility with the use of a bronchodilator is defined as an increase in FEV1 of 12% or 200 ml [19, 20, 40].

The aim of this study was to determine the effect of double combined therapy of inhaled corticosteroid and long-acting beta agonist - ICS/LABA and triple combined therapy ICS/LABA plus LTRA: leukotriene receptor antagonist (Montelukast) in patients with uncontrolled severe persistent asthma by analyzing of IL-13 and FEV1 before and after 6 months of therapy.

## Material and Methods

We have examined 56 patients with a diagnosis of uncontrolled severe persistent asthma treated at the Clinic of Pulmonology and Allergology. The available subjects were divided at random into two groups. First group with 27 patients were treated with combined therapy of ICS/LABA (500/50 mcg-twice daily). Second group with 29 patients were treated with ICS/LABA (500/50 mcg-twice daily) plus Montelukast (10 mg-daily).

In each of them were measured serum IL-13 levels by the ELISA method before the treatment and after 6 months of therapy at the Institute of Immunobiology and Human Genetics, Faculty of Medicine, Ss Cyril and Methodius University of Skopje. The reference values for IL-13 are 0 to 6.9 pg/ml. Spirometry was done with spirometer Schiller SP-1 according to the standard methodology at the Clinic of Pulmonology and Allergology, Faculty of Medicine, Ss Cyril and Methodius University of Skopje. We were analyzed FEV1- Forced expiratory volume in the first second.

### Inclusion Criteria:

uncontrolled severe persistent asthma patients. The classification was according to the actual version of the GINA guidelines (Global Initiative for Asthma) [1] and Guidelines for the Diagnosis and Management of Asthma (EPR-3) of National Asthma Education Prevention Program (NAEPP) [19]. The age of the patients was 18-70 years.

### Exclusion criteria:

pregnancy, severe diseases of the immune, endocrine, haematological cardiac, renal, gastrointestinal, neurological system, psychiatric disorders, and neoplastic diseases.

### Statistical analysis

The results were statistically analyzed according to the Wilcoxon Pairs Test. The significances value were taken p < 0.05 and a highly significant p < 0.01.

## Results

First group with 27 patients were treated with combined therapy of ICS/LABA (500/50 mcg-twice daily). The average serum levels of IL-13 before the treatment were 907.37 ± 601.95 pg/ml and after 6 months treatment were 527.47 ± 577.25 pg/ml. In this group were registered highly significant difference in the values of IL-13 before and after the treatment for a period of 6 months (G1: Z=3.19; p=0.0014) (Table 1).

**Table 1:** Serum concentration of IL-13 in asthma patients treated with ICS/LABA (reference values 0 to 6.9 pg/ml)

Therapy ICS/LABA	Mean	Std. Dev.	Min-Max	Median	25-75 Percentiles
Before the treatment	907.37	601.95	49.36-2067.32	787.3	368.77-1416.12

After 6 months	527.47	577.25	0.16-2356.53	350.32	18.19-974.12

Wilcoxon Pairs Test, Z = 3.19; p = 0.0014.

In this group the average values of FEV1 before the treatment were 37.33 ± 10.57%, and after 6 months treatment they were increased on 57.33 ± 13.27%. Statistically, this difference of 12.00 and is highly significant (p < 0.001), with significantly increased the values of FEV1.

**Table 2:** The descriptive statistics – FEV1 in asthma patients treated with ICS/LABA

Therapy ICS/LABA	Mean	Std. Dev.	Min-Max	Median
Before the treatment	37.33	10.57	18.0 – 55.0	34.0

After 6 months	57.33	13.27	32.0 – 68.0	57.0

T = -12.54; p < 0.001**.

Second group with 29 patients were treated with ICS/LABA (500/50 mcg-twice daily) plus Montelukast (10 mg-daily). Before the start of therapy with ICS/LABA (500/50 mcg-twice daily) plus Montelukast (10 mg-daily), were registered much higher values of serum IL-13 (the average was 1028.21 ± 621.02 pg/ml) and after 6 months of treatment significantly reduces their value (the average was 252.21 ± 427.23 pg/ml), Z = 4.7; p < 0.001 (Table 3).

**Table 3:** Serum concentration of IL-13 in asthma patients treated with ICS/LABA plus Montelukast (reference values 0 to 6.9 pg/ml)

Therapy ICS/LABA plus Montelukast	Mean	Std.Dev.	Min-Max	Median	25-75 percentiles
Before the treatment	1028.21	621.02	67.35-2444.65	757.15	623.27-1514.29

After 6 months	252.21	427.23	0.4-427.23	81.04	2.97-217.72

Wilcoxon Pairs Test Z = 4.7; p < 0.001**.

The patients who were treated with a combination of ICS/LABA and Montelukast, average values of FEV1 before the treatment were 36.76 ± 10.26, and after 6 months therapy were 51.75 ± 11.74. The difference in the average value before and after therapy 14.99 confirmed as statistically highly significant (p < 0.001) (Table 4).

**Table 4:** The descriptive statistics – FEV1 in asthma patients treated with ICS/LABA plus Montelukast

Therapy ICS/LABA plus Montelukast	Mean	Std. Dev.	Min-Max	Median
Before the treatment	36.76	10.26	17.0-53.0	38.0

After 6 months	51.75	11.74	32.0-73.0	53.0

T = -10.125, p < 0.001**.

## Discussion

The goal of asthma treatment is to achieve and maintain clinical total control. Combination therapy with ICS/LABA represents the gold standard in the treatment of asthma, safe and effective treatment which is recommended by the Global Initiative for Asthma in the treatment of uncontrolled severe persistent asthma in the step 3 and 4. Combination high dose of ICS/LABA, generally provides additional benefit but it is well recognized that not in all patients will achieve well-controlled asthma despite an appropriately high dose of ICS or ICS/LABA combination therapy, in such patients, there is a need for additional add-on therapy such as treatment with a LTRA. Addition of a leukotriene receptor antagonist (LTRA) to patients who have considered to be insufficiently controlled resulted in a significant clinically improvement in asthma control, pulmonary function and quality of life [1, 19, 21-24].

In our study serum IL-13 was increased in all patients before the treatment. After 6 mounts therapy with combination of ICS/LABA in patients with uncontrolled severe persistent asthma we found decreases of serum IL-13, but added the Montelukast in this combination proved superior effect and allow achievement of total control of asthma with subjective clinical improvement. Unfortunately there are not available studies that analyze the level of IL -13 from the effect of adding of montelukast to combination therapy ICS/LABA in patients with uncontrolled severe persistent asthma.

It is known that the significant elevations in IL-13 are found in the airways of patients with both allergic and nonallergic asthma [24]. In the study of Shimbori C, Montelukast treatment also decreased mRNA levels of IL-6, IL-10, IL-13, and TGF-β1, all of which were elevated in fibrotic lungs [26].

In several studies have confirmed that Montelukast add-on therapy with ICS or ICS/LABA treatment is effective for managing asthma in achieving control and reducing asthma symptoms in patients who were previously uncontrolled asthma symptoms with ICS or ICS/LABA treatment who had uncontrolled [27-29].

In our study the value of FEV1 at the start of therapy were lower in both groups and after 6 months of therapy confirmed as statistically significant improvement of lung function, increase especially in the group which used Montelukast and ICS/LABA.

That is referenced in other studies when added to ICS, Montelukast induced further improvement in symptoms and pulmonary function, particularly in patients still symptomatic despite treatment with ICS. Although in short-term studies the combination of ICS plus LABA was more effective on symptoms and pulmonary function than ICS plus Montelukast [30-34].

Virchow et al in a recent article from an observational study on 1681 patients not controlled by ICS, or ICS plus LABA, showed that the addition of Montelukast improved both asthma control and asthma-related quality of life [21, 34].

In a 6-week study in symptomatic patients on high-dose ICS and short-acting b2-agonists, the addition of an LTRA significantly improved lung function and reduced exacerbations. In addition, in a smaller, open label study of 313 patients with insufficiently controlled patients on fixed combination therapy with ICS and LABAs, Dupont et al. observed an improvement in asthma symptoms and pulmonary function with add-on LTRA therapy after 2 months of therapy [21, 35, 36].

Many long-term studies on large groups of adult and pediatric patients, showed clinical and laboratory safety profile for Montelukast because no reported significant or considerable side effects observed for placebo or active control/usual care therapies. The safety profile of Montelukast did not change with long-term [36-39].

The relatively small number of participants may be considered a limitation factor.

In conclusion, treatment with ICS/LABA plus Montelukast proved to be superior compared to therapy of ICS/LABA in patients with uncontrolled severe persistent asthma, leading to reduced inflammation and allows achievement of well controlled of asthma with subjective clinical improvement. The reduced values of IL-13 after 6 month treatment with ICS/LABA led to clinical improvement, achieving good control of the disease and certainly able to reduce the dose of ICS/LABA. This suggests that IL-13 is an important marker for monitoring of evolution and success of therapy in patients with asthma.

In the future, research in the field of therapy in asthma, may be directed to discover a new molecules which specifically inhibited the activity of Th2 cytokines, which actually indirectly reduce mediators and markers of inflammation.
